# The Case of the Infection that Wasn't !

**DOI:** 10.4103/0974-777X.56246

**Published:** 2009

**Authors:** Jennifer Eatrides, Veronica T Tucci, Richard Schrot

**Affiliations:** *University of South Florida College of Medicine, Tampa, Florida*

**Keywords:** Abdominal pain, Eosinophilic esophagitis, Gastroesophageal reflux disease

## Abstract

Eosinophilic esophagitis is an under-recognized inflammatory disorder of the esophagus. It has been frequently diagnosed in pediatric patients; however, over the last few years, there has been an increase in the number of cases recognized in adults as well. Despite this fact, eosinophilic esophagitis (EE) is often a delayed diagnosis in the primary care setting due to the overlapping symptoms it shares with other esophageal and gastrointestinal disorders such as gastroesophageal reflux disease and gastroenteritis, as well as a lack of awareness among physicians who see adult patients. We performed an exhaustive search of the literature, which revealed over 400 articles on EE; however, most were reported in gastroenterology or autoimmune specialty journals. We report a case of eosinophilic esophagitis in a 39-year-old man who presented with persistent epigastric abdominal pain and who was diagnosed via endoscopy and biopsy.

## INTRODUCTION

Eosinophilic esophagitis (EE) is a disorder of the esophagus characterized by infiltration of eosinophils in the esophageal mucosa.[1] Adult patients typically present with dysphagia and food impaction; however, chest pain, abdominal pain, diarrhea, weight loss and gastroesophageal reflux disease (GERD) symptoms have also been reported.[1] Although most early literature has shown EE to be prevalent in children, a recent study using a national database found the largest proportion of patients to be between 30 and 40 years of age.[2] The exact mechanism of EE remains unclear. However, patients with a history of asthma or atopy appear more susceptible.[3,4] Although the majority of patients presenting with EE show esophageal rings or strictures upon endoscopy, it is not uncommon for esophageal abnormalities to be absent, and a high index of suspicion should be maintained particularly in those patients who have not responded to traditional treatment regimens for GERD. Further complicating the diagnosis of EE is that fact that many physicians may opt not to do a biopsy on a seemingly healthy esophagus. A diagnosis of EE, however, depends on biopsy results.[5] Many of the presenting symptoms of EE are also characteristic of GERD, which contributes to the often delayed diagnosis of these patients.[1] A key feature of EE that distinguishes it from GERD is the amount of eosinophilic infiltration of the esophagus (GERD having <10 eosinophils (eso)/High Powered Field (HPF) and EE having ≥15 eos/HPF).[1,3,5]

## CASE REPORT

A 39-year-old man presented with nausea, diarrhea and epigastric abdominal pain of 4 to 5 days in duration with loss of appetite. The patient was out of work for several days and was unable to resolve his symptoms despite the use of pantoprazole (Protonix^®^) and had lost 4 pounds since his symptoms first presented. He described his pain as a dull, constant ache, resembling “hunger pains” or “knots in the stomach.” His pain worsened when he sat upright or performed activities such as driving. The patient also mentioned that his mother had experienced similar symptoms, though a diagnosis had never been made. The patient had no prior history of atopy or asthma. Indeed, his past medical history was unremarkable.

Upon physical examination, the patient was found to have epigastric abdominal tenderness when palpated. Normal bowel sounds were present, and the exam did not reveal any other concerns.

Over the course of a month following his initial presentation, the patient returned several times with persistent epigastric abdominal pain. Although his nausea, vomiting and diarrhea were resolved, his epigastric abdominal pain persisted. Physical examinations consistently found him to have epigastric abdominal tenderness when palpated, with no other abnormalities.

When pantoprazole alone did not resolve the patient's epigastric pain after 2 weeks, he was prescribed lansoprazole (Prevacid^®^), taken once daily. After this did not relieve the pain, lansoprazole was increased to twice daily. Unfortunately, even this increased dosing did not resolve our patient's pain. Prevacid was discontinued and replaced with oral esomeprazole magnesium (Nexium^®^) 40 mg twice daily. Although the patient experienced slight improvement with Nexium^®^, he continued to experience tenderness in the epigastric region.

Complete blood count test (CBC), Comprehensive metabolic panel (CMP), lipid panel, amylase, serum lipase and *Helicobacter pylori* antibody IgG tests were performed. His laboratory tests revealed hyperlipidemia, as well as elevated serum glucose, total cholesterol and LDLs, but were otherwise unremarkable. A CT scan of the abdomen with and without contrast was performed and found to be unremarkable except for a unilateral pars defect on the left at L5 and a subcentimeter Boxniak type I simple renal cyst of the right kidney. These findings were considered to be unrelated to his presenting symptoms.

**Figure 1:**
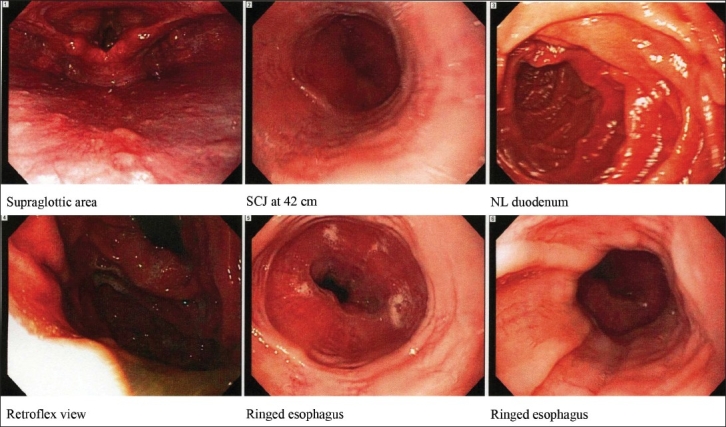
Concentric rings in the distal esophagus, consistent with eosinophilic esophagitis

Endoscopy revealed concentric rings in the distal esophagus and gastritis in the antrum [Figure 1]. The presence of concentric rings visualized during endoscopy prompted the gastroenterologist to do a biopsy on the esophagus as well. A cold-forceps biopsy specimen was taken from the esophagus, and multiple fragments were received in formalin. Two cold-forceps biopsy specimens were taken from the stomach for urease test to determine whether gastritis was caused by *Helicobacter pylori.*

**Figure 2:**
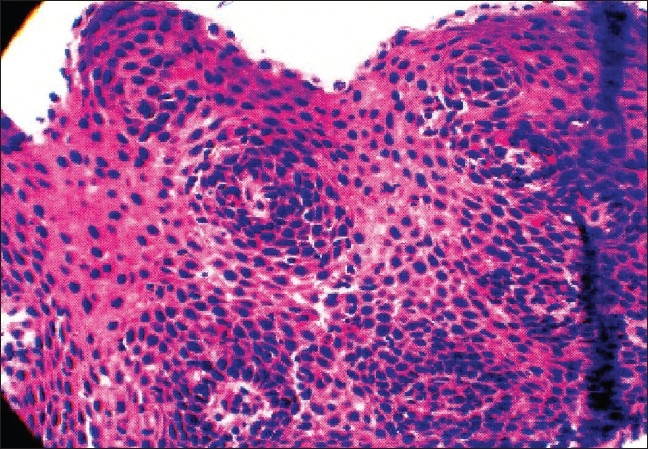
Esophageal squamous mucosa showing elongation of submucosal papillae, basal cell hyperplasia and prominent (>20/HPF) intraepithelial eosinophils. No dysplasia or malignancy is seen. No glandular epithelium is seen. No viral inclusions are identified. No yeast, fungi or other pathogenic organisms are seen

A negative Campylobacter Like Organism (CLO) test confirmed the absence of *H. pylori.* The esophageal specimens were visualized. The pathology report showed esophageal squamous mucosa with elongation of submucosal papillae, basal cell hyperplasia and prominent (>20/ high powered field (HPF)) intraepithelial eosinophils [Figure 2]. Based on the biopsy, the diagnosis was consistent with eosinophilic esophagitis.

Following a diagnosis of EE, the patient's symptoms resolved with a 3-month course of fluticasone as well as the continuation of Prevacid^®^ 30 mg with meals to avoid upset stomach. After 1 month, the patient reported resolution of his epigastric abdominal pain.

## DISCUSSION

Eosinophilic esophagitis is an increasingly recognized finding in adult patients. The differential diagnosis for EE is extensive and includes many common conditions such as GERD and gastroenteritis. We have summarized the clinical presentation and laboratory and imaging/ endoscopy findings in Table 1 to allow the practitioner to better differentiate some of the more common diagnoses from EE. Indeed, prior to early 90s, esophageal eosinophilia was often assumed to be associated with GERD, regardless of the number of intraepithelial eosinophils per HPF.[1,6] It is now accepted that although eosinophils may also be present in GERD, numbers are markedly increased in EE (>15 eos/HPF). Although recognition of EE has increased over the last few years, studies have shown that diagnosis is still often delayed even when highly suggestive features are present.[1] Studies have shown that the incidence of EE is higher than that of other well-recognized disorders, yet somehow, it is the diagnosis that is often missed.[7] Because of the focus on pediatric patients in early literature, providers who deal exclusively with adult patients are less likely to recognize the significance of marked eosinophilia in esophageal samples.[8] Eosinophilic esophagitis has been shown to be more prevalent in males, is present on all continents except Africa, does not seem to favor specific ethnicities, and has a typical adult-onset age between 20 and 40 years.[1,4]

**Table 1:** Differential diagnosis of eosinophilic esophagitis

Diagnosis	Clinical presentation	Laboratory values, histopathology, imaging/endoscopy
GERD	Heartburn, regurgitation, dysphagia, chest pain, water brash, globus sensation, odynophagia, nausea	≤5 eos/HPF
Ulcer/gastritis	GI bleeding, early satiety, dysphagia, weight loss, unexplained anemia	Urease-positive biopsy
Gastroenteritis	abdominal pain, diarrhea, GI bleeding, colitis, malabsorption	GI symptoms, peripheral eosinophilia, elevated serum IgE
Esophageal web/stricture	Solid food dysphagia	Ringed esophagus
Achalasia	Dysphagia (solids and liquids), difficulty belching, weight loss, regurgitation, chest pain, heartburn, globus sensation, hiccups	Dilated esophagus with residual material, normal mucosa
Pancreatitis	Abdominal pain, nausea, vomiting, pancreatic insufficiency, fat malabsorption, pancreatic diabetes	Imaging studies may show calcium deposits
Eosinophilic esophagitis	Dysphagia, abdominal pain, vomiting, food impaction	Ringed esophagus, white exudates, >15 eos/HPF

Adults with eosinophilic esophagitis typically present with dysphagia or food impaction; however, chest pain, abdominal pain, diarrhea and weight loss have also been reported.[1,4,9,10] The frequencies of symptoms of EE have been summarized in Table 2. Because the symptoms are so similar to those of GERD, proton pump inhibitors are usually the first line of treatment attempted, and are typically ineffective.[3,8] In the present case, the patient lacked the hallmark symptoms of dysphagia or food impaction; however, he presented with abdominal pain, diarrhea and weight loss that were resistant to treatment with Prevacid alone. Although these symptoms are noted to be present in adults and children with EE, adult cases more often present with dysphagia whereas epigastric abdominal pain, vomiting and diarrhea are more characteristic of presenting symptoms in pediatric cases.[1] The lack of dysphagia in our patient can possibly be attributed to his lack of allergies; it is suggested that food allergies correlate with presence of dysphagia.[2] Our patient's seemingly atypical adult presentation of EE may have contributed to his delayed diagnosis.

**Table 2:** Frequency of symptoms of eosinophilic esophagitis[1–5,8,10,11,12,15]

Symptom	Frequency %
Dysphagia	29-100
Nausea/vomiting	2.5-28
Food impaction	25-100
Acid reflux	2-64
Chest pain	1-58
Abdominal pain	3-40
Peripheral eosinophilia	10-50
Elevated IgE	52-69
Food allergies	4-27
Mucosal rings	55-69
Linear furrows	33-86
Seasonal allergies	43-52
Asthma	23-50
Normal esophagus	7

Although the mechanism of EE remains unclear, it is important to note that eosinophils are not present in the esophagus under normal circumstances. In the case of EE, eosinophils, T cells and mast cells are all involved in the inflammation, which is localized to the esophagus.[4] EE closely resembles the chronic airway inflammation observed in bronchial asthma in terms of its clinical, histological and immunopathogenic features — the correlation between EE and patients with food allergies and asthma suggests that EE occurs as a result of an allergic or immunologic sensitization.[4,9,14] A 30-year study by Prasad *et al.* in Olmstead County showed diagnosis dates predominantly in late summer/ early fall in a group with seasonal allergies and/ or asthma, indicating a correlation between EE and inhaled aeroallergens.[11] The same group also reported increased incidence of EE over the last 30 years and found it to be a relapsing disease in a significant number of patients — with a median of 4.2 years till recurrence.[11] The exact etiology of EE is unknown; however, TH2 cytokines (IL-5 and IL-13), eotaxin and TNF-α play a role in eosinophil recruitment, proliferation and pathogenesis within the esophageal mucosa.[12,13] The presence of TH2 cytokines indicates that EE occurs via a TH2 type immunological reaction.[3,14] This is important in terms of possible treatment options that may provide a more targeted approach towards the treatment of EE.

In addition to the correlation between allergies and EE, it has also been suggested that there is a familial component as well. A pediatric study in Hamilton County involving 103 patients found 3 sibling pairs to be affected; the mother of one sibling pair also had a diagnosis of EE.[7] This indicates a genetic component to EE and may aid in the diagnosis of cases where a familial pattern can be identified.

Endoscopic features may predict eosinophilic esophagitis, but a diagnosis is dependent on histological findings of ≥15 eos/HPF.[5,8] Peripheral eosinophilia is present in about half of the patients presenting with EE and gives an indication as to the severity — patients with increased peripheral eosinophilia were shown to have more attacks of dysphagia.[4] Although EE cases typically show a concentric ringed esophagus, as presented in this case, endoscopic clues are not always so clear. A study by Pasha *et al.* showed 17% of patients in a 212-patient study to have normal-appearing esophagus but were confirmed to have eosinophilic esophagitis.[3] In the case presented here, the patient exhibited endoscopic features indicative of EE, which were confirmed by a biopsy specimen revealing squamous mucosa basal cell hyperplasia and prominent (>20 eos/HPF) intraepithelial eosinophils [Figures 1 and 2].

Eosinophilic esophagitis can be managed by several different means, the most common of which is through treatment with corticosteroids.[3,5] We have summarized common treatment modalities below in Table 3. A review of literature showed that esophageal dilation, restricted diets, antihistamines, systemic and topical corticosteroids, and leukotriene receptor antagonists have all been suggested as possible treatments; however, the use of corticosteroids has remained the standard. A 3-year study of topical corticosteroid treatment for EE showed EE to be a chronic relapsing condition that is similar to asthma. They suggested that although fluticasone does improve the initial symptoms of EE, the relapse rate of 69%, needing repeated treatment, emphasizes the need for repeated treatment with fluticasone.[15] Potential therapies aim to prevent further migration of eosinophils into the esophageal mucosa through antibodies against IL-5, which is known to be involved in eosinophil recruitment; however, this treatment is not approved at this time.[1,12] Infliximab, a potent inhibitor of TNF-α, has also been suggested because of the critical role that TNF-α plays in the pathogenicity of EE; a pilot study showed that 1 of 3 patients achieved a partial response.[13] A recent review suggested that acid suppression is useful both as a diagnostic tool and as treatment in combination with corticosteroid treatment, the effects of which may be attributable to an increased sensitivity to acid in patients with EE.[1] Our patient was treated with fluticasone and Prevacid and, as previously mentioned, reported complete resolution of his epigastric abdominal pain at his 1-month follow-up visit. The patient has remained symptom free for 2 years since his initial diagnosis.

**Table 3:** Treatment modalities for Eosinophilic esophagitis[12,15,16]

Treatment	Rationale
Elimination diet	Elimination of foods with food allergens from diet
Elemental diet	Solid foods are replaced with synthetic amino acid solution; foods are gradually added back to determine reactivity
Systemic corticosteroids	Anti-inflammatory, improve symptoms, long-term maintenance required
Topical corticosteroids	Anti-inflammatory, improve symptoms with fewer systemic side effects, longterm maintenance required
Leukotriene inhibitor (Monteleukast)	Blocking leukotrienes reduces eosinophilic migration
IL-5 antibodies	IL-5 mediates eosinophil accumulation in tissues; studies ongoing
Endoscopic dilation	Manages strictures and food impaction, transient treatment

Eosinophilic esophagitis should be considered in patients that present with persistent epigastric abdominal pain and show little improvement with use of proton pump inhibitors. Because a diagnosis definitive of EE can only be made after histological confirmation, it is important to collect biopsy specimens at the time of endoscopy in patients presenting with symptoms characteristic of EE. With increased awareness among family physicians, internists and emergency physicians, this disorder, commonly misdiagnosed as *H. pylori* infection, can be more quickly identified in patients, who may then receive appropriate treatment.
